# Data mining techniques for predicting acute kidney injury after elective cardiac surgery

**DOI:** 10.1186/cc10951

**Published:** 2012-03-20

**Authors:** J Van Eyck, J Ramon, F Guiza, G Meyfroidt, M Bruynooghe, G Van den Berghe

**Affiliations:** 1 K.U. Leuven, Heverlee, Belgium

## Introduction

Development of acute kidney injury (AKI) during the postoperative period is associated with increases in both morbidity and mortality. The aim of this study is to develop a statistical model capable of predicting the occurrence of AKI in patients after elective cardiac surgery.

## Methods

A total of 810 adult (>18 years) elective cardiac surgery patients, admitted to the surgical ICU of the University Hospital of Leuven between 18 January 2007 and 8 January 2009, were retrospectively selected for this study. Patients with an ICU stay of less than 24 hours, as well as patients suffering from chronic kidney disease, were excluded. Relevant patient records were extracted from an electronic database system and analyzed using data mining techniques [1]. The main advantage of these techniques is that they are capable of automatically selecting the variables that are relevant to a particular problem. Using such a data mining algorithm, predictive models were built on a development cohort of 385 patients and validated on a separate cohort of 425 patients.

## Results

In this study, two separate models were developed for predicting the occurrence of AKI (defined as RIFLE stage three or need for renal replacement therapy) within a week after the patient's admission. An initial model was built using only readily available admission data (including demographic information, previous treatments and pre-admission values for physiological variables). This resulted in an AUC of 0.6056 (95% CI, 0.4874 to 0.7239) on the validation cohort. The initial model was then extended by adding information on administered medication, measurements of physiological parameters and laboratory results available during the first four hours of the patient's ICU stay. This new model resulted in an AUC of 0.8339 (95% CI, 0.7364 to 0.9315) on the validation cohort.

## Conclusion

In this study, we have shown that data mining techniques are a viable option for developing predictive models in a clinical setting. Furthermore, we have shown that by adding information gathered during the patient's stay, a model's performance can drastically improve compared to a model using only admission data. Thus, it might be possible to further improve existing scoring systems such as the Thakar score [2] and the simplified renal index [3].
